# Phytochrome A Protects Tomato Plants From Injuries Induced by Continuous Light

**DOI:** 10.3389/fpls.2019.00019

**Published:** 2019-01-30

**Authors:** Aaron I. Velez-Ramirez, Dick Vreugdenhil, Frank F. Millenaar, Wim van Ieperen

**Affiliations:** ^1^Horticulture and Product Physiology, Wageningen University & Research, Wageningen, Netherlands; ^2^Laboratory of Plant Physiology, Wageningen University & Research, Wageningen, Netherlands; ^3^Centre for Biosystems Genomics, Wageningen, Netherlands; ^4^Monsanto Holland B.V., Bergschenhoek, Wageningen, Netherlands

**Keywords:** continuous light, *Solanum lycopersicum*, tomato, phytochrome, far-red light, photosynthesis down-regulation

## Abstract

Plants perceive and transduce information about light quantity, quality, direction and photoperiod via several photoreceptors and use it to adjust their growth and development. A role for photoreceptors has been hypothesized in the injuries that tomato plants develop when exposed to continuous light as the light spectral distribution influences the injury severity. Up to now, however, only indirect clues suggested that phytochromes (PHY), red/far-red photoreceptors, are involved in the continuous-light-induced injuries in tomato. In this study, therefore, we exposed mutant and transgenic tomato plants lacking or over-expressing phytochromes to continuous light, with and without far-red light enrichment. The results show that *PHYA* over-expression confers complete tolerance to continuous light regardless the light spectrum. Under continuous light with low far-red content, PHYB1 and PHYB2 diminished and enhanced the injury, respectively, yet the effects were small. These results confirm that phytochrome signaling networks are involved in the induction of injury under continuous light.

**HIGHLIGHTS:**

- *PHYA* over-expression confers tolerance to continuous light regardless the light spectrum.
- In the absence of far-red light, PHYB1 slightly diminishes the continuous light-induced injury.
- Continuous light down-regulates photosynthesis genes in sensitive tomato lines.

## Introduction

Sunlight is essential for life on earth. Plants not only have developed a photosynthetic machinery to harvest the energy from the sun but also a set of photoreceptors that can sense light quality, quantity, direction and photoperiod, allowing them to adjust their growth and development according to the light environment (Christie, 2007; Jiao et al., 2007; Bae and Choi, 2008; Moglich et al., 2010). When artificial light is used, however, physiological disorders can arise, yet it is not always clear whether the cause resides in the photosynthetic or light sensing processes. A remarkable example, first reported by Arthur et al. (1930), are the injuries that domesticated tomatoes develop when exposed to continuous light (CL), which include mottled chlorosis and poor photosynthetic performance. It is still unclear if the altered photosynthetic performance is a cause or consequence, and which of the light signaling mechanisms plays a role, if any. Based on evidence accumulated over the years and a modern understanding of plant physiology, we previously proposed that both, photosynthesis and light signaling might play a role in the development of the injury in CL-grown tomato plants (Velez-Ramirez et al., 2011).

The languished CL-tolerance found in wild tomato species (Daskaloff and Ognjanova, 1965) was mapped to the *type III Light harvesting chlorophyll a/b binding protein* 13 gene (*LHCB type III CAB-13* or *CAB-13*) on chromosome seven (Velez-Ramirez et al., 2014). In addition to genetic mapping, expression data showed a positive correlation between *CAB-13* expression and CL-tolerance. For instance, in CL-sensitive tomatoes, CL down-regulated *CAB-13* expression, but in a *Solanum lycopersicum* CL-tolerant introgression line, known as “CLT,” *CAB-13* expression was high under CL. Furthermore, when *CAB-13* expression was silenced in the CLT line, using virus-induced gene silencing (VIGS), plants lost their tolerance to CL (Velez-Ramirez et al., 2014). This evidence strongly suggests the involvement of the photosynthetic machinery in the CL-induced injury. Previous studies also reported that the light spectral distribution influences the severity of the injury (Arthur, 1936; Globig et al., 1997; Murage et al., 1997; Demers and Gosselin, 2002). For instance, Demers and Gosselin (2002) showed that a higher percentage of blue light increased the CL-induced injury, and Globig et al. (1997) showed that addition of far-red light reduced the injury in tomato. In addition, Murage et al. (1997) reports different degree of CL-induced injury when eggplants (same genus as tomato, *Solanum melongena*) were exposed to different light spectral distributions. Hence, the potential involvement of light signaling pathways must not be discarded. Hence, from all currently known photoreceptors (Moglich et al., 2010), phytochromes are the most likely candidates to be involved, yet direct evidence is missing.

Phytochromes (PHY) translate red and far-red light into biological signals thanks to covalently attached chromophores that enable photo-conversion between two forms: the Pr form and the biologically active Pfr form. With a peak absorbance for red light, Pr is converted to Pfr upon light absorption. In turn, far-red light most effectively transforms back the Pfr to the biologically inactive Pr form (Rockwell et al., 2006; Bae and Choi, 2008). Light colors other than red and far-red also drive Pr/Pfr inter-conversion at a lower efficiency, and the absorbance and quantum yield varies for different wavelengths. Thus, in photoequilibrium, the ratio of Pr/Pfr depends on the light spectral distribution (Sager et al., 1988). Upon photoisomerization, Pfr translocates to the nucleus where it activates the degradation of phytochrome-interacting factors (PIFs) and inhibits two E3 ubiquitin ligases (CUL4-DDB1-COP1-SPA and CUL4-DDB1-DET1-COP10); this results in light responses within the plant as PIF transcription factors promote dark and shade responses, and several positive light regulators, like Elongated Hypocotyl 5 (HY5), are degraded by COP1- and DET1-containing E3 ubiquitin ligases (Bae and Choi, 2008; Chen and Chory, 2011; Leivar and Quail, 2011; Lau and Deng, 2012).

Arabidopsis has five phytochromes, PHYA to PHYE (Sharrock and Clack, 2002). Phytochromes are classified in two types, the PHYA and PHYB branch, yet this dichotomy does not directly correlates with their molecular properties and functions across species (Bae and Choi, 2008). Unlike PHYB, PHYA can only translocate to the nucleus with the help of Far-red Elongated Hypocotyl 1 (FHY1) and FHY1-like (FHL) (Chen and Chory, 2011; Casal, 2013). Phytochrome-mediated, light-induced responses are classified into three categories, namely very-low-fluence response (VLFR), low-fluence response (LFR) and high-irradiance response (HIR; Casal et al., 1998).

The tomato genome also encodes five phytochromes known as PHYA, PHYB1, PHYB2, PHYE and PHYF (Hauser et al., 1995). Little is known about PHY functions in adult tomato plants. Most of the studies have focused on germination, anthocyanin biosynthesis and seedling de-etiolation. Anthocyanin biosynthesis in tomato is regulated by a PHYB1/PHYB2-dependent red-HIR component as well as PHYA-dependent far-red-HIR and a non-far-red-reversible red-LFR component (Kerckhoffs et al., 1997; Weller et al., 2000); however, PHYA antagonizes the effect of PHYB1, and PHYB2 cannot compensate for the loss of PHYB1 as most of the red-HIR depends on PHYB1 (Weller et al., 2000). In addition, unlike other species, in tomato such PHYA-dependent red-LFR of anthocyanin biosynthesis is strongly reduced in the *phyB1phyB2* double mutant (Weller et al., 2000). Regarding other light-regulated processes, mutant studies show that the red-HIR component of tomato seedling de-etiolation depends, redundantly, on PHYB1 and PHYB2, yet, as with anthocyanin biosynthesis, only PHYB1 can compensate for the loss of PHYB2 (Weller et al., 2000). However, transgenic over-expression of *PHYB2* not only fully compensates but also enhances the red-HIR component of anthocyanin biosynthesis even in the *phyB1phyB2* double mutant background (Husaineid et al., 2007). Over-expression of PHYB1 had little or no effect on red-HIR responses (Husaineid et al., 2007). Unlike anthocyanin biosynthesis, seedling de-etiolation is not affected by PHYA (Weller et al., 2000). Recently, silencing of tomato *PHYE* showed that, in the absence of PHYB1 and PHYB2, PHYE is required for the shade avoidance response (Schrager-Lavelle et al., 2016). All together, phytochromes in tomato, as in other species, not only interact differently to control various traits but also in response to different light wavelengths and fluence rates. This makes it crucial to test the effect of each PHY under different light environments for every trait of interest.

In this study, in order to test whether phytochromes play a role in the CL-induced injuries in tomato, we exposed several phytochrome mutants and over-expressing lines to CL with two contrasting far-red light contents. The results show that *PHYA* over-expression confers complete CL-tolerance regardless the light spectral distribution. The roles of PHYB1 and PHYB2 appear to be less dominant than PHYA and depend on the light spectral distribution. These results not only confirm that phytochrome signaling networks are involved in the injury induction under CL but also provide further clues in understanding why CAB-13 is so important in determining tolerance/sensitivity to CL.

## Materials and Methods

### Plant Materials and Light Treatments

Tomato phytochrome mutants (*phy*) and over-expressing (*PHYOE*) lines are all in the *Solanum lycopersicum* cv. Moneymaker background, which is continuous light (CL) sensitive (Velez-Ramirez et al., 2014). All lines used here have been described previously: *phyA*-null mutant [*phyA-1* (*fri^1^*)] (van Tuinen et al., 1995b); *phyB1*-null mutant [*phyB-1* (*tri^1^*)] (van Tuinen et al., 1995a); *phyB2*-null mutant [*phyB2-1* (70F)] and *phyB1phyB2* double mutant (Weller et al., 2000); and *PHYAOE* (A/3), *PHYB1OE* (B1/4) and *PHYB2OE* (B2/9) transgenic lines (Husaineid et al., 2007). Some lines carried a circadian-clock reporter construct (VQ2) consisting of the *Luciferase* gene behind the *Cab* promoter (*CAB::Luciferase*) (Personal communication, van der Krol, 2014). Lines marked with an ^∗^ carry the *Cab::Luciferase* construct. This construct had no effect on the phenotype of tomato plants grown under all light treatments. In addition, the presence, pattern and severity of chlorosis were not affected by the circadian-clock reporter construct. Although mutant *phyB2*^∗^ and over-expressing lines *PHYB1OE*^∗^ and *PHYB2OE*^∗^ were only available in our seed bank with the *Cab::Luciferase* construct, the results showed that these lines are comparable to lines lacking the construct.

**FIGURE 1:**
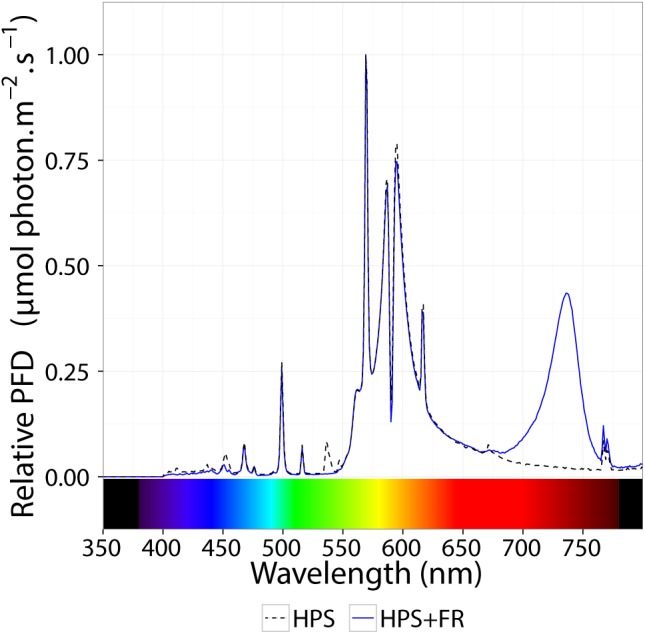
Relative spectral distribution of the light treatments. The dashed black line represents the spectral distribution of high–pressure sodium lamps (HPS). The blue line represents the spectral distribution HPS light supplemented with far-red light-emitting diodes. See Table 1 for extra details.

Plants were grown in rockwool blocks at 21°C and 70% RH. Commercial hydroponic nutrient solution for tomato was used (Yara Benelux B.V., Vlaardingen, Netherlands); after combining and diluting premixed liquid fertilizers, the solution contained 12.42 mM NO_3_, 7.2 mM K, 4.1 mM Ca, 3.34 mM SO_4_, 1.82 mM Mg, 1.2 mM NH_4_, 1.14 mM P, 30 μM B, 25 μM Fe, 10 μM Mn, 5 μM Zn, 0.75 μM Cu, and 0.5 μM Mo (EC = 2.00 dS m^-1^ and pH = 5.0–5.5). Supplemented with incandescence lamps (Philinea T30 120W, Philips, Eindhoven, Netherlands), high-pressure sodium (HPS) lamps (Master SON-T Green Power 400W, Philips, Eindhoven, Netherlands) were installed above a double ceiling. The photosynthetically active photon flux density (PPFD) was 345 μmol m^-2^ s^-1^. Red-to-far-red ratio was 2.89, and the phytochrome photostationary state (PSS; Sager et al., 1988) was 0.858. After growing the plants for 2 weeks under 16 h photoperiod, plants were transferred to continuous light with and without the addition of far-red (FR) light. For the HPS+FR light, Philinea incandescent lamps were also used, yet double number of HPS lamps was installed and a neutral density (ND) film (Film 209 0.3ND, LEE Filters, Hampshire, United Kingdom) filtered ∼50% of the visible light. Above ∼700 nm, nonetheless, filter transmittance was slightly higher, contributing to the enrichment of FR light. To increase even further the FR light, two types of far-red light-emitting diodes (LEDs) were placed below the ND filter, namely (GreenPower LED production module FR 120, Philips, Eindhoven, Netherlands) and (Orean Retrofit Far-red LED, Lemnis Lighting B.V., Barneveld, Netherlands). Finally, after placing the plants 15 cm closer to the lamp, a homogeneous PPFD of 344 μmol m^-2^ s^-1^ was reached; the red-to-far-red ratio and PSS were 0.18 and 0.662, respectively. Figure 1 shows the resulting spectral distribution of HPS and HPS+FR light; see Table 1 for further details.

**Table 1:** Light treatment characteristics.

Light treatment	PFD (μmol photons m^-2^ s^-1^)	PPFD (μmol photons m^-2^ s^-1^)	PSS (Sager et al., 1988)	R:Fr ratio	Irradiance (W m^-2^)	Photoinhibitory activity $\overset{800}{\underset{nm=400}{\Sigma }}\alpha ɸ$ (Jones and Kok, 1966)
High pressure sodium + Philinea (HPS)	368	345	0.86	2.89	74	15.4
High pressure sodium + Philinea + Far Red (HPS + FR)	504	344	0.66	0.18	96	14.9


### Chlorophyll Fluorescence Imaging

Imaging of the maximum quantum efficiency of photosystem II (*F*_v_/*F*_m_) (Baker, 2008) was performed as previously described (Velez-Ramirez et al., 2014). In summary, intact leaflets (attached to the plant) were dark-adapted using dark adapting clips (Li-Cor Biosciences, Lincoln, NE, United States). After 20 min of dark adaptation, leaflets were detached and immediately used for measurements in a chlorophyll fluorescence imaging system (FluorCam 800MF, Photon System Instruments, Brno, Czechia). Leaflet average *F*_v_/*F*_m_ was calculated using ImageJ software version 1.44o (Schneider et al., 2012). The *F*_v_/*F*_m_ parameter assessed the presence and severity of CL-induced injury as previously reported (Velez-Ramirez et al., 2014, 2017a,b). For clarity, nonetheless, results are also expressed as ΔΔ*F*_v_/*F*_m_. The Δ*F*_v_/*F*_m_ values represent the response of phytochrome mutants and over-expressing lines to the light treatments taking the average CL-induced decrease of *F*_v_/*F*_m_ in the wild type (Moneymaker, MM) as a reference and correcting for the average slight decrease in *F*_v_/*F*_m_ observed under 16-h photoperiod. That is,

$$\Delta \Delta F_{v}/F_{m}=-\left(\left[\overset{¯}{MM_{CL}}-\left\{\overset{¯}{mu\,\tan t_{CL}}\,\;or\,\;\overset{¯}{OE_{CL}}\right\}\right]-\left[\overset{¯}{MM_{16h}}-\left\{mu\,\tan t_{16h\cdot }\,or\,\;\overset{¯}{OE_{16h}}\right\}\right]\right).$$

Where $\overset{¯}{{MM}_{CL}}$ and $\overset{¯}{{MM}_{16h}}$ are the average *F*_v_/*F*_m_ in the wild-type Moneymaker under CL and 16-h photoperiod, respectively; $\overset{¯}{{mu\,\tan t}_{16h}}$, $\overset{¯}{{OE}_{16h}}$ or $\overset{¯}{{mu\,\tan t}_{CL}}$ are the average *F*_v_/*F*_m_ in each mutant or overexpressing line under 16-h photoperiod or CL.

### Mapping of RNAseq Data to Tomato-Specific KEGG Pathways

Previously published expression data of CL-sensitive A131 and CL-tolerant CLT tomato plants exposed to CL (Velez-Ramirez et al., 2014) was mapped to tomato-specific KEGG pathways. The Sol Genomic Network/Ensembl gene identifiers (*e.g*., Solyc07g063600.2) of the original data set were mapped to UniProt accessions (*e.g*., K4CH43) and then to the KEGG/GENEID/Entrez IDs (*e.g*., 101268123) using the UniProt ID mapping tool^1^; only genes mapping to unique IDs were used. From the 31350 genes in the original data set, 14219 had a unique mapping between all IDs. The R package “Pathview” (Luo and Brouwer, 2013) was used to map the originally reported LogFold change to the following tomato-specific KEGG pathways: “Photosynthesis” (sly00195), “Photosynthesis antenna proteins” (sly00196) and “Porphyrin and chlorophyll metabolism” (sly00860). For nodes containing more than one gene, mean LogFold change was used.

### Statistical Analysis

Statistical significance of the leaflet average *F*_v_/*F*_m_ was determined with an *ANOVA* test performed with IBM SPSS Statistics software version 19 (IBM, Somers, NY, United States).

## Results

**FIGURE 2:**
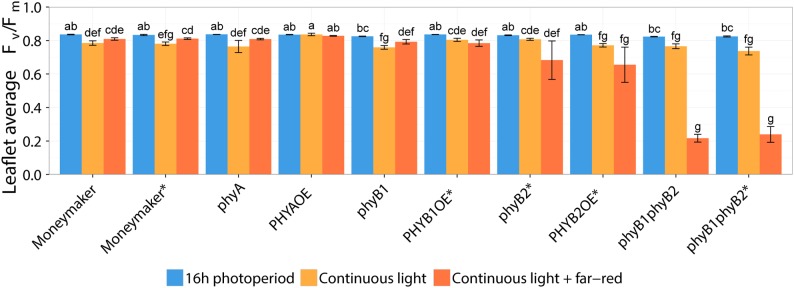
Effect of two continuous light treatments on phytochrome mutants and over-expressing tomato lines. Tomato plants were grown under 16-h photoperiod for 2 weeks and then transferred to continuous light provided by high-pressure sodium (HPS) lamps (Continuous light, orange bars) or HPS lamps supplemented with far-red light (Continuous light + far-red, red bars). Control plants were kept at 16-h photoperiod (16 h photoperiod, blue bars). After 2 weeks, chlorophyll fluorescence imaging assessed the level of continuous light-induced injury. Leaflet average *F*_v_/*F*_m_ values represent mean ± SE (*n* = 4); bars not sharing the same letter are statistically different (*P* < 0.05). Lines marked with an ^∗^ carry a circadian clock reporter construct (*Cab::Luciferase*).

**FIGURE 3:**
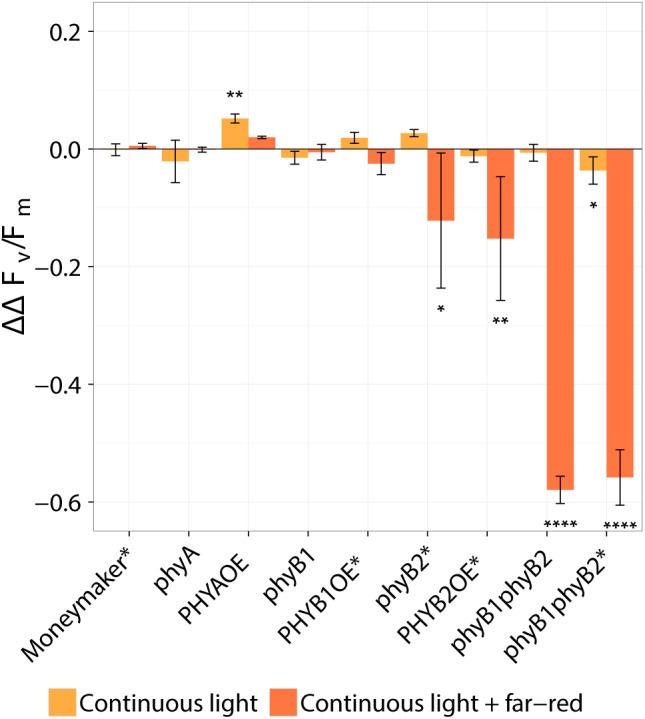
Effect of continuous light on tomato, taking into account the pleiotropic effect of phytochrome mutations observed under 16-h photoperiod. Tomato plants were grown under 16-h photoperiod for 2 weeks and then transferred to continuous light provided by high-pressure sodium (HPS) lamps with and without far-red light enrichment (orange and red bars, respectively). The ΔΔ*F*_v_/*F*_m_ values represent the response of several phytochrome mutants and over-expressing lines to the light treatments taking the average continuous-light-induced decrease of *F*_v_/*F*_m_ in the wild type (Moneymaker, MM) as a reference and correcting for the average slight decrease in *F*_v_/*F*_m_ observed under 16-h photoperiod. That is, ΔΔF_v_/F_m_ = -([$\overset{¯}{{MM}_{CL}}$ - {$\overset{¯}{{mu\,\tan t}_{CL}}$ or $\overset{¯}{{OE}_{CL}}$}] - [$\overset{¯}{{MM}_{16h}}$ - { ${mu\tan t}_{16h}¯$ or $\overset{¯}{{OE}_{16h}}$}]. Where $\overset{¯}{{MM}_{CL}}$ and $\overset{¯}{{MM}_{16h}}$ are the average *F*_v_/*F*_m_ in the wild-type Moneymaker under 16-h photoperiod and continuous light, respectively; $\overset{¯}{{mu\,\tan t}_{16h}}$, $\overset{¯}{{OE}_{16h}}$, $\overset{¯}{{mu\,\tan t}_{CL}}$, or $\overset{¯}{{OE}_{CL}}$ are the average *F*_v_/*F*_m_ in each mutant or overexpressing line under 16-h photoperiod or continuous light. The minus at the beginning of the equation is just for clarity; a positive value represents a positive effect of each phytochrome mutation or overexpression. Values represent mean ± SE (*n* = 4). Asterisk on top of error bars indicate that the ΔΔ*F*_v_/*F*_m_ value is statistically different from zero; ^∗^*P* < 0.05, ^∗∗^*P* < 0.01, and ^∗∗∗∗^ *P* < 0.0001. See original data on Figure 2 Lines marked with an ^∗^ carry a circadian clock reporter construct (*Cab::Luciferase*).

**FIGURE 4:**
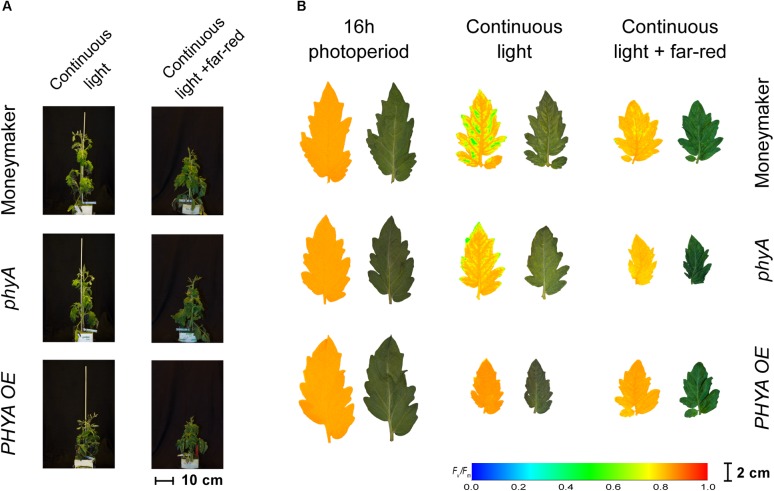
Phenotypes of phytochrome A mutant (*phyA*) and over-expressing line (*PHYA OE*) under continuous light. Both lines are in the moneymaker background. In panel **(A)**, tomato plants were grown under 16-h photoperiod for 2 weeks and then exposed to continuous light or continuous light + far-red for 2 weeks. In panel **(B)**, representative leaflets (topmost fully expanded leaf) of plants in previous panel. Photographs (right side) and chlorophyll fluorescence images (left side) show the appearance of chlorosis on some lines. Across leaflet surface, a false color scale indicates *F*_v_/*F*_m_ value (see guide at the bottom-left corner). See Figure 2, 3 for *F*_v_/*F*_m_ means across treatments.

Two-week-old plants of tomato wild-type, *phy mutants* and *PHYOE* lines were exposed to continuous light (CL) provided by high-pressure sodium (HPS) light, with and without addition of far-red light. Control plants were kept under 16-h photoperiod provided by HPS without supplemental far-red light. The presence and severity of CL-induced injury was assessed by the chlorophyll fluorescence parameter *F*_v_/*F*_m_ (Velez-Ramirez et al., 2014, 2017a,b). The parameter *F*_v_/*F*_m_ represents the maximum quantum efficiency of photosystem II (PSII), and a value of 0.83 is remarkably constant in non-stressed plants across species (Baker, 2008); therefore the lower the *F*_v_/*F*_m_ value, the higher the injury is. After 2 weeks of treatment, all tomato lines grown under 16-h photoperiod showed *F*_v_/*F*_m_ values that were not significantly different from the wild-type Moneymaker plants (*P* > 0.05), and were similar to those reported in the literature for non-stressed healthy leaves (Baker, 2008). Under non-injurious conditions, leaflets of *phyB1* and *phyB1phyB2* single and double mutants had slightly lower *F*_v_/*F*_m_ values than the wild-type (Figure 2). For clarity and to account for these pleiotropic effects, results are also expressed as ΔΔ*F*_v_/*F*_m_, which is the difference between *F*_v_/*F*_m_ of wild-type Moneymaker and the mutant/over-expressing lines under CL, after correction for the *F*_v_/*F*_m_ value observed under 16-h photoperiod in the corresponding line (Figure 3). A positive ΔΔ*F*_v_/*F*_m_ value represents a positive effect of the phytochrome mutation or over-expression on the CL-induced injury, *i.e*., a reduction in CL injury. Under CL, Moneymaker had lower leaflet *F*_v_/*F*_m_ value (*P* < 0.05) than under 16-h light (Figure 2) and displayed clear interveinal chlorosis (Figure 4). Raw data used to make Figure 2, 3 is available in Supplementary Table 1.

### *Phytochrome A* Over-Expression Protects Tomato Plants From Continuous Light

Interestingly, over-expression of *PHYA* completely prevented CL-induced injury regardless of the light spectrum and the *F*_v_/*F*_m_ values under CL did not significantly differ from that of plants grown under 16-h photoperiod (*P* > 0.05) (Figure 2). Figure 3 shows a positive effect in *F*_v_/*F*_m_ value, in *PHYA* over-expression line, after considering pleiotropic effects. Figure 4 shows that *PHYAOE* leaflets exposed to CL for 2 weeks showed no signs of chlorosis, while wild-type Moneymaker leaflets did. Supportive of a protective effect of PHYA, *phyA* mutant plants had slightly lower *F*_v_/*F*_m_ values than Moneymaker under CL (Figure 2). Overall, the results suggest that PHYA signaling diminishes the injuries that CL induces in tomato plants.

### Double Mutant *phyB2phyB2* Shows Mottled Chlorosis Even Under 16 h Photoperiod

Under CL with far-red, both *phyB2*^∗^ and *PHYB2OE*^∗^ lines have lower *F*_v_/*F*_m_ values than wild-type Moneymaker^∗^ (Figure 2, 3), suggesting that PHYB2 is involved in the CL-induced injury in tomato, at least when far-red light is enriched.

**FIGURE 5:**
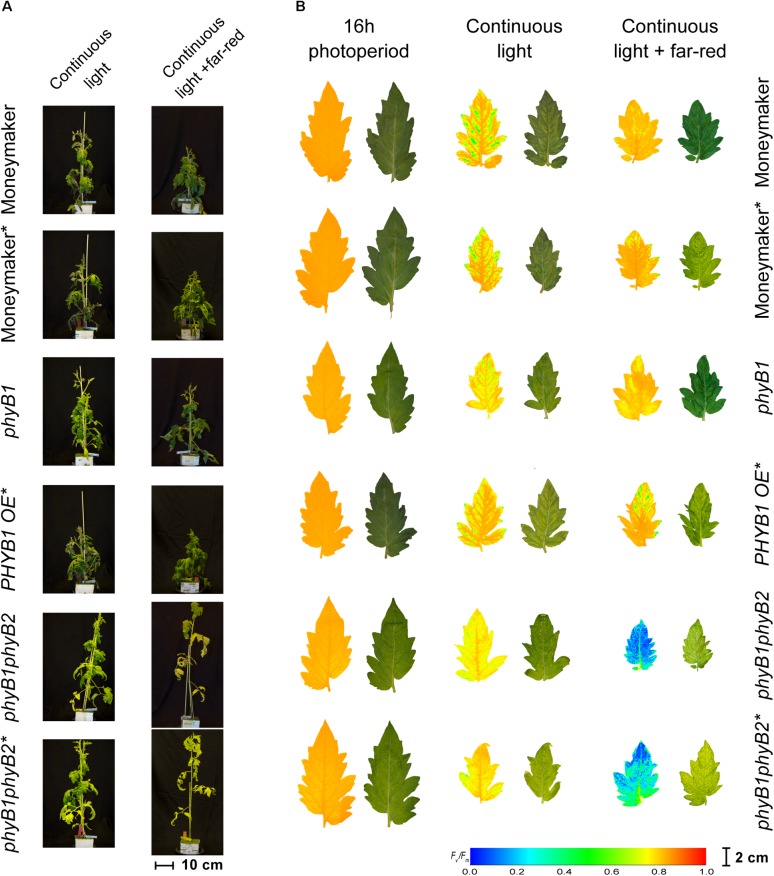
Phenotypes of phytochrome B mutants and over-expressing lines under continuous light. Phytochrome B1 and B2 mutants (*phyB1* and *phyB2*), over-expressing lines (*PHYB1 OE* and PHYB2 OE) and double B1B2 mutant (*phyB1phyB2*). All lines are in the moneymaker background. In panel **(A)**, tomato plants were grown under 16-h photoperiod for 2 weeks and then exposed to continuous light or continuous light + far-red for 2 weeks. In panel **(B)**, representative leaflets (topmost fully expanded leaf) of plants in previous panel. Photographs (right side) and chlorophyll fluorescence images (left side) show the appearance of chlorosis on some lines. Across leaflet surface, a false color scale indicates *F*_v_/*F*_m_ value (see guide at the bottom-left corner). In both panels, lines marked with an ^∗^ carry a circadian clock reporter construct (*Cab::Luciferase*).

Interestingly, double mutant lines *phyB1phyB2* and *phyB1phyB2^∗^* showed signs of chlorosis under 16-h photoperiod (Figure 5). The mottled, interveinal chlorosis (Figure 5B) resembles the characteristic mottled chlorosis that CL-exposed tomato leaves develop. Under CL without far-red, *phyB1phyB2* and *phyB1phyB2^∗^* double mutant lines showed CL-induced injuries similar to control Moneymaker (Figure 2, 3, 5). Interestingly, when CL treatment was enriched with far-red light, the CL-induced injury in both *phyB1phyB2* double mutant lines severely increased (Figure 2, 3, 5).

### Enrichment of Far-Red Light Diminishes Continuous-Light-Induced Injury in Tomato

In contrast to the *phyB1phyB2* double mutant lines, Moneymaker leaves showed less CL-induced injury symptoms when exposed to CL enriched with far-red light, compared with plants exposed to continuous HPS light without far-red enrichment, reflected in higher *F*_v_/*F*_m_ values (Figure 2). This is consistent with previous reports (Globig et al., 1997). Although the differences are not significant, this trend is also visible in the chlorophyll fluorescence images (Figure 5). Interestingly, *phyA* mutants showed the same trend as the wild type; that is less CL-induced injury when CL is enriched with far-red light (Figure 2, 4), suggesting that the protective effect of far-red light does not depend on PHYA, or that other phytochrome may compensate in the absence of PHYA.

### Expression of PHYB Is Lower in Continuous-Light-Sensitive Plants Than in Tolerant Ones

In order to assess the effects of CL on tomato *PHY* expression, we mined a published whole transcriptome RNAseq dataset (Velez-Ramirez et al., 2014) derived from tomato plants exposed to CL at the same PPFD and spectral distribution (HPS) as in the preset study. This data set contains differential expression levels for two contrasts; the first contrast compares the effect of CL on the CL-sensitive tomato inbred line A131, and the second one evaluates the differences under CL between A131 and a CL-tolerant introgression line named CLT (Velez-Ramirez et al., 2014). In A131 plants, CL exposure resulted in a significant up-regulation of *PHYE* expression, while the differential expression of all other *PHYs* is not significant (Table 2). Under CL, the expression of *PHYB1* and *PHYE* is higher in CL-tolerant CLT than in CL-sensitive A131 plants. Table 2 also shows the differential expression of *HY5*, a transcription factor downstream of the photoreceptors (Jiao et al., 2007; Lee et al., 2007). Interestingly, *HY5* is significantly up-regulated by CL in A131 tomato plants, but there is no difference in expression between A131 and CLT plants when both are exposed to CL.

**Table 2:** Expression ratio of tomato *phytochrome* (*PHY*) genes and *HY5* transcription factor between A131 16 h vs. A131 24 h and A131 24 h vs. CLT 24 h^§^.

		A131 24 h–A131 16 h				A131 24 h–CLT 24 h				
			
Gene	Name	logFC	logCPM	*P*-value	FDR-corrected *p*-value	logFC	logCPM	*P*-value	FDR-corrected *p*-value
Solyc10g044670.1	*Phytochrome A* (*PHYA*)	0.40	5.14	0.06	0.19	0.24	5.14	0.25	0.60
Solyc01g059870.2	*Phytochrome B1* (*PHYB1*)	–0.11	5.45	0.59	0.80	–0.65	5.45	0.00	0.03
Solyc05g053410.2	*Phytochrome B2* (*PHYB2*)	–0.50	2.49	0.16	0.39	–0.67	2.49	0.05	0.26
Solyc02g071260.2	*Phytochrome E* (*PHYE*)	–1.21	4.90	0.00	0.00	–0.68	4.90	0.00	0.03
Solyc07g045480.2	*Phytochrome F* (*PHYF*)	0.23	3.57	0.31	0.59	–0.19	3.57	0.41	0.75
Solyc08g061130.2	*Elongated Hypocotyl 5* (*HY5*)	0.90	1.55	0.01	0.03	0.52	1.55	0.09	0.36
Solyc07g063600.2	*CAB-13*	–3.43	11.61	1.83E-12	1.17E-10	–2.1	11.61	5.27E-6	3.21E-4


*^§^ A131 and CLT are sensitive and tolerant to continuous light (CL), respectively. Data taken from Velez-Ramirez et al. (2014).*

### Continuous Light Induces Photosynthetic Down-Regulation in Tomato Plants

Considering that the CL-induced injury is proposed to be photosynthetic down-regulation (Velez-Ramirez et al., 2014, 2011), and phytochromes regulate the expression of several photosynthetic genes in tomato (Peters et al., 1998), we also evaluated the effect of CL on photosynthesis at the transcriptional level. Hereto, the gene expression level of both contrasts was mapped to tomato-specific KEGG pathways (Supplementary Figures 1, 2). As species-specific KEGG pathways are drawn over standard KEGG maps, care should be taken with the interpretation of white nodes. Some nodes contain no expression information because (i) that specific node does not exist in tomato (*e.g*., bacterial antenna proteins in Supplementary Figure 2), (ii) the node is not yet annotated in tomato (only ∼25,000 tomato genes are currently annotated in the KEGG database), and/or (iii) the gene(s) associated to that node were not detected in the data set (only ∼14,000 genes with KEGG annotation were detected in this data set). Additionally, the expression level of each node might be the mean of several genes; in Supplementary Figure 2, for example, *LHCB1* color-coded expression is the average fold change of the six type I LHCB proteins annotated in the KEGG database. For an overview of annotated tomato genes in KEGG pathways, follow the links in the figure legends.

Supplementary Figure 1 shows the tomato KEGG pathway for “photosynthesis” (sly00195) as affected by CL. Interestingly, CL down regulates most of the annotated genes in A131 tomato plants. When both A131 and CLT plants were exposed to CL, most of the annotated genes showed lower expression in the CL-sensitive A131 plants than in CLT, which is CL-tolerant. Similarly, Supplementary Figure 2 shows the tomato KEGG pathway for “photosynthesis antenna proteins” genes (sly00196) as affected by CL. In A131 plants, CL represses the expression of all antenna proteins genes of PSII (*LHCB*) and PSI (*LHCA*) (Supplementary Figure 2). Additionally, CLT plants showed higher expression of all *LHCB* and *LHCA* proteins than A131 plants when both are exposed to CL. In other words, most of the photosynthesis genes in CL-sensitive tomato plants exposed to CL are expressed at lower levels than in both control plants under 16-h photoperiod and CL-tolerant plants exposed to CL. Furthermore, the same trend is observed in the tomato KEGG pathway for “porphyrin and chlorophyll metabolism” (sly00860) (Supplementary Figures 3, 4). That is, all enzymes in the chlorophyll biosynthesis pathway are expressed at lower levels in CL-exposed A131 plants compared with A131 plants under 16-h photoperiod and CLT plants exposed to CL. Altogether, CL down regulates photosynthesis in CL-sensitive but not in CL-tolerant tomato plants.

## Discussion

At an irradiance of 345 μmol m^-2^ s^-1^, the *PHYAOE* line was clearly tolerant to continuous light (CL) regardless of the light spectral distribution (Figure 2–4). This PHYA-dependent CL-tolerance in tomato is active at high light; in contrast to the classic VLFR and far-red HIR attributed to PHYA, which are active at much lower light intensities (Casal, 2013). Light induces the degradation of the PHYA Pfr form and attenuates PHYA signaling by repressing Far-red elongated hypocotyl 1 (FHY1) and FHY1-like (FHL), which are needed to shuttle PHY to the nucleus (Chen and Chory, 2011; Casal, 2013). Tomato PHYA is also degraded by light; after exposing wild-type and *PHYAOE* tomato seedlings to red light at 3 μmol m^-2^ s^-1^, PHYA was greatly reduced, but not eradicated, as quantified with antibodies (Husaineid et al., 2007). Light-grown *phyA* and *PHYAOE* tomato plants show phenotypes different from wild-type plants (van Tuinen et al., 1995b; Husaineid et al., 2007). In most cases, this can be hypothetically attributed to PHYA accumulated during the daily dark period, which is subsequently activated to the Pfr form at dawn. Supporting the classic role of PHYA in the VLFR, *PHYAOE* tomato seedlings showed the strongest phenotypic differences from wild-type under continuous red light at ∼0.01 μmol m^-2^ s^-1^ as observed in fluence rate response curves of anthocyanin biosynthesis (Husaineid et al., 2007). In Arabidopsis, however, a PHYA-dependent red HIR has been suggested as the use of *phy* mutant combinations showed that PHYA, PHYB and PHYD redundantly regulate mature plant architecture under continuous red light at an intensity of 160 μmol m^-2^ s^-1^. At 100 and 180 μmol m^-2^ s^-1^ of continuous red light, *phyB* mutants displayed inhibition of hypocotyl elongation, yet *phyAphyB* double mutants were remarkably insensitive to red light (Franklin et al., 2007). An irradiance-dependent photoprotection of nuclear PHYA is proposed to explain such PHYA-dependent red HIR as PHYA was rapidly degraded after 2 h but was still detectable for up to at least 8 h of red light exposure at 1 or 180 μmol m^-2^ s^-1^, respectively, and nuclear-localized PHYA:YFP epifluorescence was still detectable after 90 min of red light at 200 and not at 1 μmol m^-2^ s^-1^ (Franklin et al., 2007). In tomato, *phyA* mutants showed anthocyanin accumulation similar to wild type under continuous red light (λ 680 nm) at ∼20 μmol m^-2^ s^-1^, but under ∼200 μmol m^-2^ s^-1^, anthocyanin content in *phyA* mutants was approximately 38% lower than in wild type (Husaineid et al., 2007), suggesting the existence of a PHYA-dependent red HIR also in tomato. Hence, we propose that the protective effect of *PHYA* overexpression against CL at high irradiances truly depends on PHYA signaling.

PHYA signaling mediates VLFRs, which are extremely sensitive to Pfr as even pulses of far-red or “green safe” light can elicit enough Pfr to saturate VLFR; PHYA also mediates light signaling during dark-to-light transitions and far-red HIR (Casal et al., 1998; Bae and Choi, 2008). This PHYA-dependent far-red HIR is unique as no other photoreceptor can mediate responses induced by far-red light (Possart et al., 2014). Although red light is most efficient eliciting Pfr, *i.e*., increasing the PSS, far-red light is most efficient triggering PHYA-dependent responses. An antagonistic photoconversion cycling model explains the shift toward far-red in PHYA action spectrum (Possart et al., 2014). Evidence shows that, similar to other phytochromes, PHYA Pfr is the active form in signaling, yet photocycling between Pr and Pfr forms is needed to both bind and, once inside the nucleus, release the FHY1/FHL transporters (Rausenberger et al., 2011). A recent study shows that Arabidopsis PHYA directly targets numerous genes related to photosynthesis, respond to light, stress and hormones in multiple far-red-modulated processes (Chen et al., 2014). Thus, investigating the PHYA-dependent mechanism protecting tomato from CL must consider the unique properties of PHYA signaling.

In Arabidopsis, the red/far-red reversible LFR is mediated by phytochromes other than PHYA (Bae and Choi, 2008), and in tomato PHYB1 and PHYB2 mediate red HIR (Weller et al., 2000). As HPS light is rich in wavelengths in the orange-red spectrum (Figure 1), resulting in a distinctively high PSS value of 0.858 (Table 1), PHYB1 and PHYB2 signaling effects were expected under HPS light without far-red enrichment. Under such light treatment, PHYB1 and PHYB2 showed opposite roles, *phyB1* and *phyB2* mutations increased and decreased the CL-induced injury, respectively; accordingly, *PHYB1* and *PHYB2* overexpression decreased and increased the injury, respectively (Figure 2, 3, 5). Interestingly, *PHYB1* expression is not affected by CL in A131 plants, but CL-tolerant CLT plants show higher *PHYB1* expression than A131 plants when both are exposed to CL (Table 2). All together, the evidence suggests that PHYB1 signaling protects tomato plants from CL-induced injury, and PHYB2 enhances the injury.

When HPS light was enriched with far-red, the *phyB2^∗^* mutant performed worse than *phyB1*, yet the *phyB1phyB2^∗^* double mutant lines performed the worst (Figure 2, 3), suggesting that, under this light condition, PHYB1 and PHYB2 redundantly protect tomato plants from CL, yet PHYB2 seems to have a dominant role. This is contrary to what was observed in anthocyanin biosynthesis and seedling de-etiolation, where PHYB1 dominates over a redundant PHYB2 (Weller et al., 2000). Intriguingly, *PHYB1OE*^∗^ and *PHYB2OE*^∗^ lines showed increased sensitivity to CL as *phyB1* and *phyB2* mutants did (Figure 3), the effect being more severe in the case of *PHYB2*. Such apparent discrepancy between the PHYB2 role inferred from *phyB2*^∗^ mutant and *PHYB2OE*^∗^ line should be interpreted carefully as we observed large variation from plant to plant and discrepancies in inferring the role of tomato PHYB2 have been observed before. For instance (Weller et al., 2000), deduced from *phyB1*, *phyB2* and *phyB1phyB2* mutant lines that PHYB2 has a negligible effect on the red HIR in anthocyanin biosynthesis as the null mutation of *PHYB2* is only seen in the *phyB1* mutant background, yet (Husaineid et al., 2007) showed that *PHYB2* overexpression not only can compensate for the loss of *PHYB1* but can even increase the wild-type red HIR to anthocyanin biosynthesis even in the *phyB1phyB2* double mutant background.

Far-red light enrichment should decrease the PHYB1/PHYB2 active Pfr pool. Considering that the PHYA-dependent red-LFR on anthocyanin biosynthesis in tomato depends on either PHYB1 or PHYB2 (Weller et al., 2000), it should also be considered that the strong protective effect of PHYA overexpression against CL-induced injury depends, at least in part, on the supportive role of PHYB1/PHYB2. In this scenario, PHYA would be similarly active under either light treatment, but under far-red light enrichment PHYB1 and PHYB2 inactivation would be accountable for the slight loss in the ability of *PHYA* overexpression to protect tomato plants from CL (Figure 3). Hence, testing whether the protective effect observed in the *PHYAOE* line remains in the *phyB1phyB2* double mutant background is of great interest for future experiments.

Phytochrome signaling might explain the observed effect of the light spectral distribution on the severity of the CL-induced injuries in tomato (Arthur, 1936; Globig et al., 1997; Murage et al., 1997; Demers and Gosselin, 2002). This effect is complex and interacts with the light intensity (Velez-Ramirez et al., 2017a). For instance, when a 16-h photoperiod of artificial solar light at 90 μmol m^-2^ s^-1^ was extended to CL by superimposing dim red or dim blue light for 24 h day^-1^ at 10 μmol m^-2^ s^-1^, no increase in CL-injury was observed in the “dim blue” treatment, yet plants exposed to the “dim red” treatment showed a slightly more CL-induced injury. However, when a 16-h photoperiod of red and blue light (at 80 and 20 μmol m^-2^ s^-1^, respectively) was extended to CL with either red or blue light at 100 μmol m^-2^ s^-1^, the “blue night” treatment was slightly more injurious that the “red night” treatment (Velez-Ramirez et al., 2017a). In other words, blue light during night was more injurious than red light, or *vice versa*, depending on the light intensity during the subjective night and/or the spectral distribution during the subjective day. Considering that tomato PHYA can mediate blue LFR in anthocyanin biosynthesis (Weller et al., 2000) and phototropism (Srinivas et al., 2004), it might be that PHYA signaling protects plants in the non-injurious “dim blue” treatment, but other factors potentially involved in the induction of injury under CL outweigh the positive effect of PHYA at higher irradiances of blue light in the “blue night” treatment. Such other factors may include photoinhibition, signaling from other photoreceptors and/or higher carbohydrate accumulation (Velez-Ramirez et al., 2011, 2017a,b).

Recently, the CL-tolerance found in wild tomato species was mapped to *CAB-13* on chromosome seven, yet the tolerance mechanism is still unclear (Velez-Ramirez et al., 2014). In this study, the discovered PHYA-dependent CL-tolerance provides additional clues as tomato *CAB-13* expression is most probably under PHYA control. This hypothesis is based on knowledge of a closely related, better-studied tomato CAB protein: CAB-1, located on chromosome two (Pichersky et al., 1985). *CAB-1* expression in tomato is up regulated by ultra violet, blue, red and far-red light (Wehmeyer et al., 1990), suggesting the involvement of several photoreceptors in its regulation. Interestingly, at least PHYA and PHYB1 regulate *CAB-1* expression in tomato (Peters et al., 1998). This suggests a link between the PHYA-mediated and CAB-13-mediated CL-tolerance in tomato, PHYA signaling might also be upstream of CAB-13 as is the case for CAB-1. In addition, RNAseq data support this hypothesis since CAB-13-mediated CL-tolerance is associated with higher expression of all tomato CAB proteins (Table 2; Velez-Ramirez et al., 2014). Actually, expression of most photosynthesis genes in CL-sensitive tomato plants is repressed by CL, while CL-tolerant plants show higher expression than sensitive plants when both are exposed to CL (Supplementary Figures 1–4). Interestingly, CL up regulates *HY5* expression in CL-sensitive tomatoes, but there is no difference between CL-tolerant and -sensitive plants (Table 2). Considering that HY5 is a positive regulator of light-responsive genes, including photosynthesis genes, it is remarkable that photosynthesis genes are down regulated while at the same time *HY5* is up regulated.

In Arabidopsis seedlings, when chloroplast development is blocked with lincomycin, HY5 is converted from a positive to a negative regulator of *LHCB1^∗^1* (Ruckle et al., 2007). Interestingly, the evidence suggests that cryptochrome 1 (CRY1) and PHYB contribute to the repression of *LHCB* when chloroplast biogenesis is blocked, yet PHYA remains as positive regulator of *LHCB* expression regardless of the chloroplast state (Ruckle et al., 2007). If a similar process occurs in tomato, it would suggest that the CL-induced injury is the result of photosynthetic down-regulation enhanced by PHYB2 and prevented by PHYB1 and PHYA. Recently, we showed that CL-induced injury is triggered by circadian asynchrony (Velez-Ramirez et al., 2017a) and correlates with starch accumulation (Velez-Ramirez et al., 2017b). PHYA can be the link between these two observations as PHYA regulates starch metabolism (Han et al., 2017) and circadian clock genes in Arabidopsis (Chen et al., 2014).

## Conclusion

Phytochrome signaling is involved in the injury induction under continuous light in tomato. *PHYA* over-expression results in complete tolerance to continuous light regardless the light spectrum. Under continuous light with low far-red content, PHYB1 and PHYB2 slightly diminished and enhanced the injury, respectively. Analysis of transcriptome data indicates that continuous light downregulates photosynthesis genes in continuous light-sensitive tomato lines but not in tolerant ones.

## Author Contributions

All authors designed the study and interpreted the data. AV-R and WvI set up the experiments. WvI secured access to tomato lines and lead the critical review of the final version. AV-R performed the measurements and data analysis and drafted the manuscript with the help of all authors.

## Conflict of Interest Statement

The authors declare that the research was conducted in the absence of any commercial or financial relationships that could be construed as a potential conflict of interest.

## Footnotes

1 www.uniprot.org/help/mapping

## Abbreviations

A131: CL-sensitive tomato line
CAB: light harvesting chlorophyll a/b-binding protein
CAB-13: *see LHCB type III CAB-13*
CL: continuous light
CLT: CL-tolerant introgression line
CRY1: cryptochrome 1
FR: far-red
*F*_v_/*F*_m_: maximum quantum efficiency of photosystem II
HIR: high-irradiance response
HPS: high-pressure sodium
HY5: elongated hypocotyl 5
LEDs: light-emitting diodes
LFR: low-fluence response
LHCA: antenna proteins of PSI
LHCB: antenna proteins of PSII
LHCB type III CAB-13 or CAB-13: type III light harvesting chlorophyll a/b-binding protein 13
MM: moneymaker tomato line
ND: neutral density
Pfr: active phytochrome form
PFD: photon flux density
PHY: phytochrome
PIFs: phytochrome-interacting factors
PPFD: photosynthetically active photon flux density
Pr: inactive phytochrome form
PSI: photosystem I
PSII: photosystem II
PSS: phytochrome photostationary state
SE: standard error
VIGS: virus-induced gene silencing
VLFR: very-low-fluence response
