# Supplementary material for: Phytochrome A Protects Tomato Plants From Injuries Induced by Continuous Light
Source: Front Plant Sci. 2019 Jan 30;10:19. doi: 10.3389/fpls.2019.00019 (PMC6363712; doi:10.3389/fpls.2019.00019)
Supplement: Supplementary file 2 [file Presentation_1.pdf]

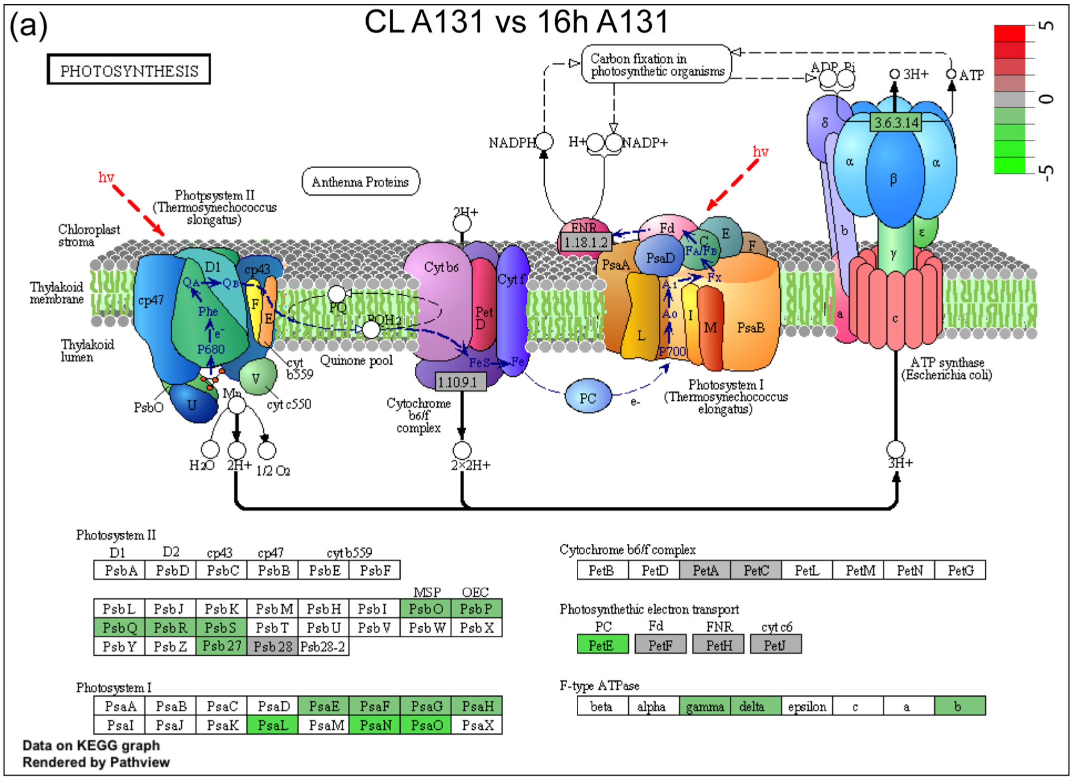

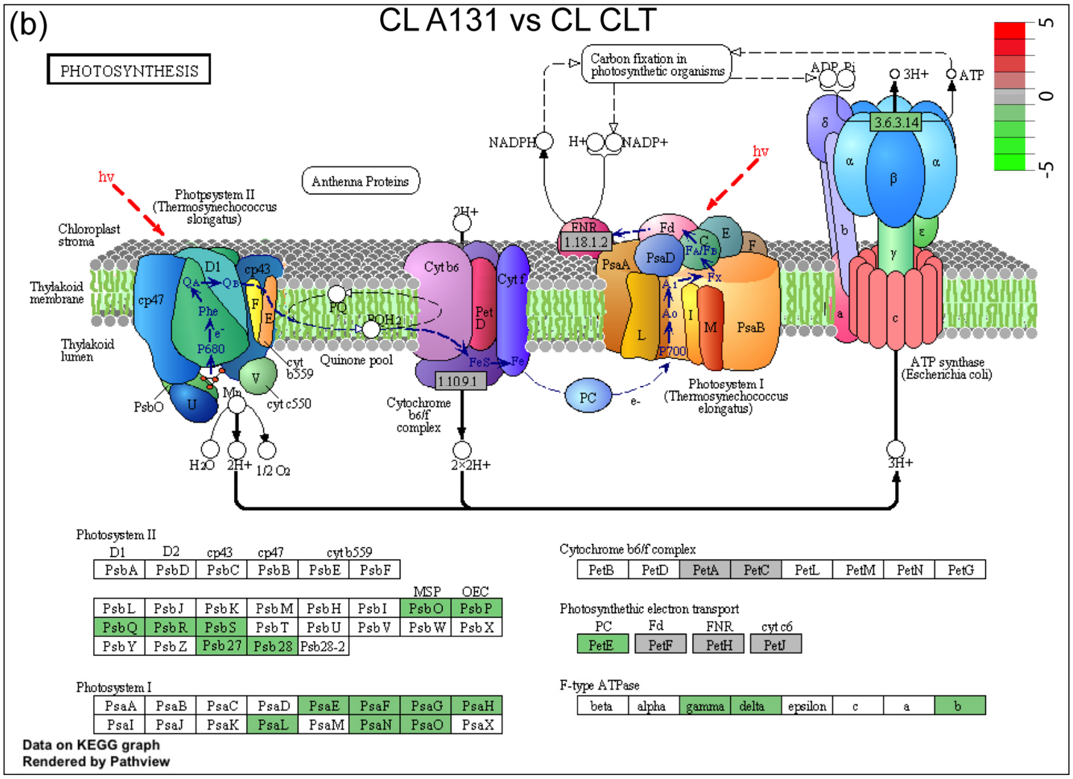

Supplementary Figure 1. Tomato “Photosynthesis” KEGG pathway as affected by continuous light. Differential expression of tomato genes in continuous light (CL)-sensitive A131 and CL-tolerant CLT tomato plants (Velez-Ramirez et al., 2014) was mapped to the tomato KEGG pathway for photosynthesis. In panel (a), contrast between A131 tomato plants exposed to 16-h photoperiod and CL. In panel (b), contrast between A131 and CLT exposed to continuous light. In both panels, each colored node represents the average Log fold change of all genes contained in that node. No expression information is available for the nodes in white because of (i) that specific node does not exist in tomato (e.g. a bacteria-specific enzyme), (ii) the node do exist in tomato, but it is not yet annotated (only ±25,000 tomato genes are currently annotated in the KEGG database) and/or (iii) the node do exist and is annotated in tomato, yet the gene(s) associated

to that node were not detected in the data set (only ±14,000 genes with KEGG annotation were detected in this data set). For detailed information on each node, visit the KEGG website on the following link: www.genome.jp/kegg-bin/show_pathway?sly00195

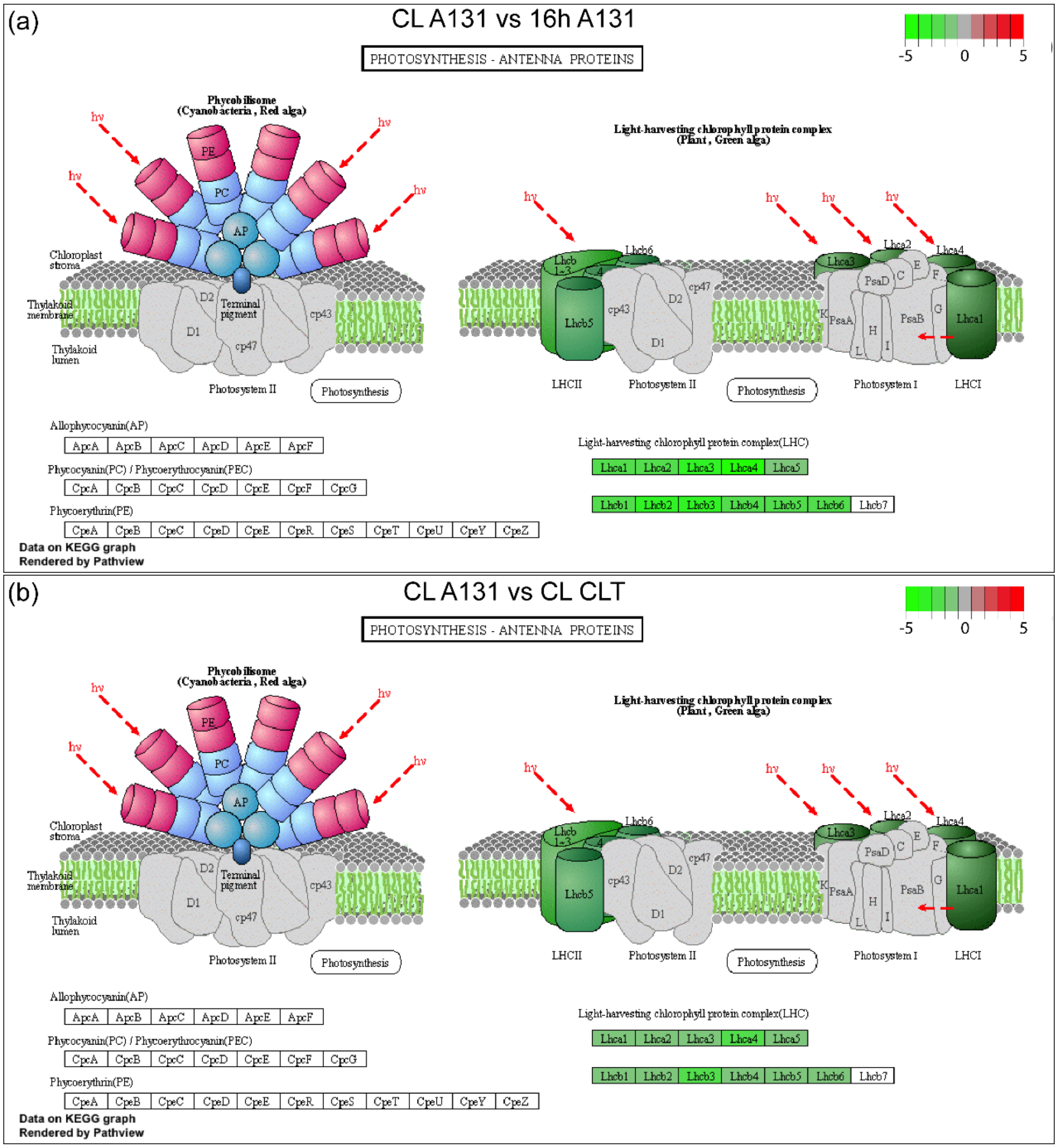

Supplementary Figure 2. Tomato “photosynthetic antenna proteins” KEGG pathway as affected by continuous light. Differential expression of tomato genes in continuous light (CL)-sensitive A131 and CL-tolerant CLT tomato plants (Velez-Ramirez et al., 2014) was mapped to the tomato KEGG pathway for photosynthetic antenna proteins. In panel (a), contrast between A131 tomato plants exposed to 16-h photoperiod and CL. In panel (b), contrast between A131 and CLT exposed to continuous light. In both panels, each colored node represents the average Log fold change of all genes contained in that node. No expression information is available for the nodes in white because of (i) that specific node does not exist in tomato (e.g. a bacteria-specific enzyme), (ii) the node do exist in tomato, but it is not yet annotated (only ±25,000 tomato genes are currently annotated in the KEGG database) and/or (iii) the node do exist and is annotated in tomato, yet the gene(s) associated to that node were not detected in the data set (only ±14,000 genes with KEGG annotation were detected in this data set). For detailed information on each node, visit the KEGG website on the following link: www.genome.jp/kegg-bin/show_pathway?sly00196

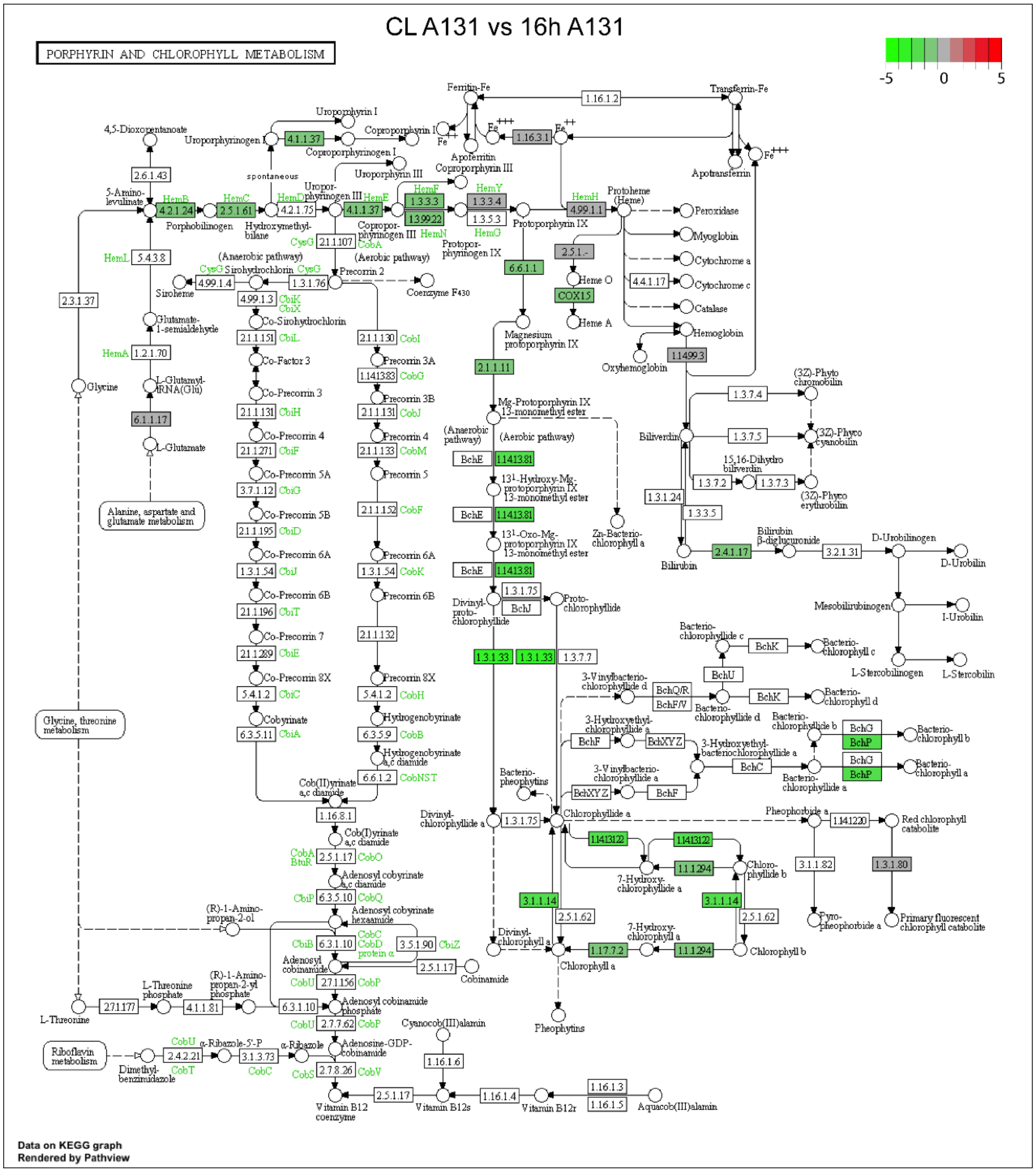

Supplementary Figure 3. Tomato "porphyrin and chlorophyll metabolism" KEGG pathway as affected by continuous light. Differential expression of tomato genes in continuous light (CL)-sensitive A131 tomato plants (Velez-Ramirez et al., 2014) was mapped to the tomato KEGG pathway for porphyrin and chlorophyll metabolism. Each colored node represents the average Log fold change of all genes contained in that node. No expression information is available for the nodes in white because of (i) that specific node does not exist in tomato (e.g. a bacteria-specific enzyme), (ii) the node do exist in tomato, but it is not yet annotated (only ±25,000 tomato genes are currently annotated in the KEGG database) and/or (iii) the node do exist and is annotated in tomato, yet the gene(s) associated to that node were not detected in the data set (only ±14,000 genes with KEGG annotation were detected in this data set). For detailed information on each node, visit the KEGG website on the following link: www.genome.jp/kegg-bin/show_pathway?sly00860

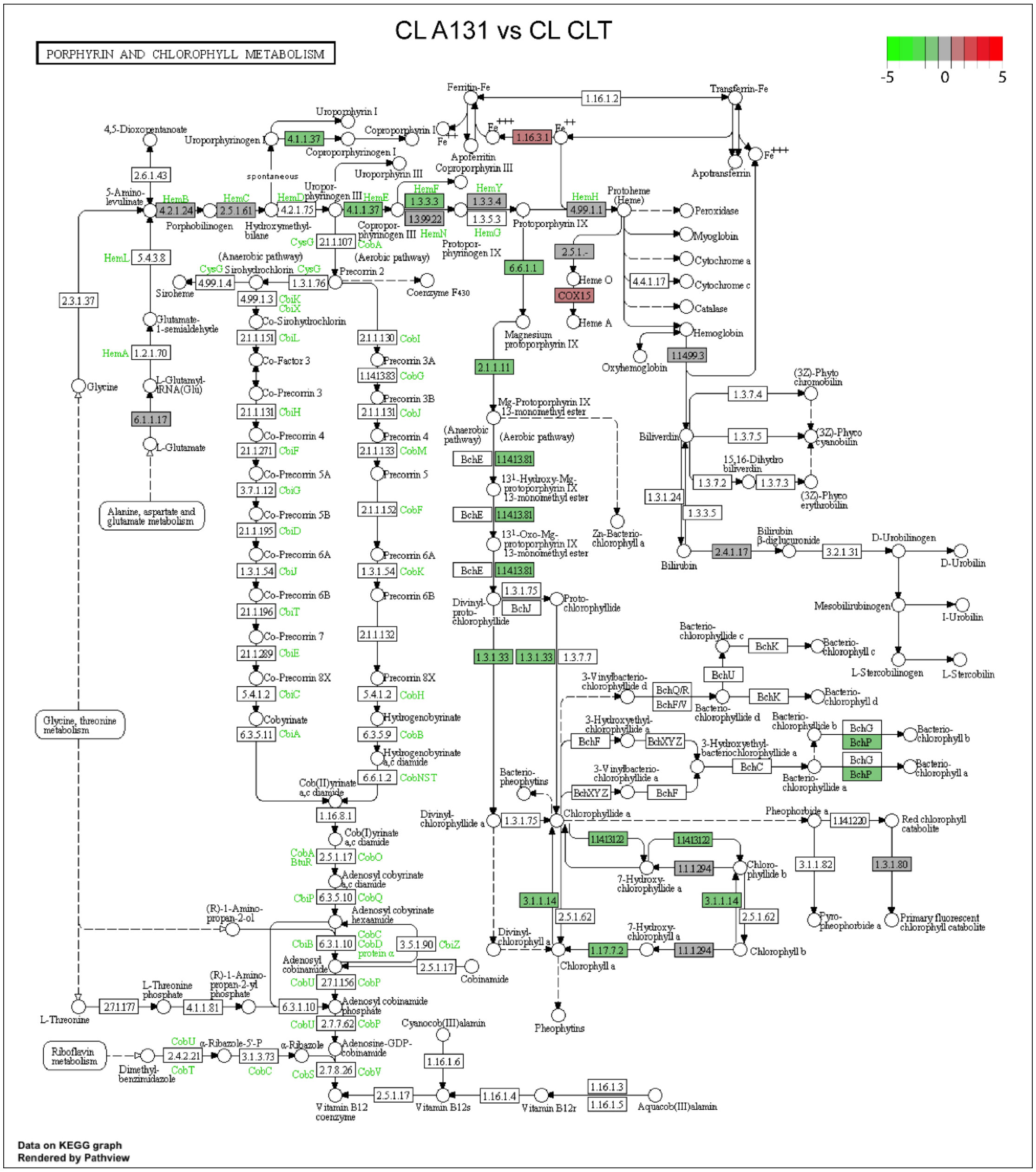

Supplementary Figure 4. Tomato “porphyrin and chlorophyll metabolism” KEGG pathway as affected by continuous-light-tolerance. Differential expression of tomato genes in continuous light (CL)-sensitive A131 and CL-tolerant CLT tomato plants (Velez-Ramirez et al., 2014) was mapped to the tomato KEGG pathway for porphyrin and chlorophyll metabolism. Both lines were exposed to CL. Each colored node represents the average Log fold change of all genes contained in that node. No expression information is available for the nodes in white because of (i) that specific node does not exist in tomato (e.g. a bacteria-specific enzyme), (ii) the node do exist in tomato, but it is not yet annotated (only ±25,000 tomato genes are currently annotated in the KEGG database) and/or (iii) the node do exist and is annotated in tomato, yet the gene(s) associated to that node were not detected in the data set (only ±14,000 genes with KEGG annotation were detected in this data set). For detailed information on each node, visit the KEGG website on the following link: www.genome.jp/kegg-bin/show_pathway?sly00860
